# Design and first baseline data of the DZNE multicenter observational study on predementia Alzheimer’s disease (DELCODE)

**DOI:** 10.1186/s13195-017-0314-2

**Published:** 2018-02-07

**Authors:** Frank Jessen, Annika Spottke, Henning Boecker, Frederic Brosseron, Katharina Buerger, Cihan Catak, Klaus Fliessbach, Christiana Franke, Manuel Fuentes, Michael T. Heneka, Daniel Janowitz, Ingo Kilimann, Christoph Laske, Felix Menne, Peter Nestor, Oliver Peters, Josef Priller, Verena Pross, Alfredo Ramirez, Anja Schneider, Oliver Speck, Eike Jakob Spruth, Stefan Teipel, Ruth Vukovich, Christine Westerteicher, Jens Wiltfang, Steffen Wolfsgruber, Michael Wagner, Emrah Düzel

**Affiliations:** 1 German Center for Neurodegenerative Diseases (DZNE), Sigmund-Freud-Straße 27, 53127 Bonn, Germany; 2 Department of Psychiatry, Medical Faculty, University of Cologne, Kerpener Straße 62, 50924 Cologne, Germany; 3 Department of Neurology, University of Bonn, Sigmund-Freud-Straße 25, 53127 Bonn, Germany; 4 Department of Radiology, University of Bonn, Sigmund-Freud-Straße 25, 53127 Bonn, Germany; 5 German Center for Neurodegenerative Diseases (DZNE), Feodor-Lynen-Straße 17, 81377 Munich, Germany; 6 Institute for Stroke and Dementia Research, Klinikum der Universität München, Feodor-Lynen-Straße 17, 81377 Munich, Germany; 7 Department of Neurodegenerative Diseases and Gerontopsychiatry, University of Bonn, Sigmund-Freud-Straße 25, 53127 Bonn, Germany; 8 Department of Psychiatry and Psychotherapy, University of Bonn, Sigmund-Freud-Straße 25, 53127 Bonn, Germany; 9 Department of Psychiatry and Psychotherapy, Charité, Charitéplatz 1, 10117 Berlin, Germany; 10 Department of Psychiatry and Psychotherapy, Charité, Hindenburgdamm 30, 12203 Berlin, Germany; 11 German Center for Neurodegenerative Diseases (DZNE), Gehlsheimer Straße 20, 18147 Rostock, Germany; 12 Department of Psychosomatic Medicine, University of Rostock, Gehlsheimer Straße 20, 18147 Rostock, Germany; 13 German Center for Neurodegenerative Diseases (DZNE), Otfried-Müller-Straße 23, 72076 Tübingen, Germany; 14 Section for Dementia Research, Hertie Institute for Clinical Brain Research and Department of Psychiatry and Psychotherapy, University of Tübingen, Calwerstraße 14, 72076 Tübingen, Germany; 15 German Center for Neurodegenerative Diseases (DZNE), Leipziger Straße 44, 39120 Magdeburg, Germany; 16 German Center for Neurodegenerative Diseases (DZNE), Charitéplatz 1, 10117 Berlin, Germany; 17 Institute of Human Genetics, University of Bonn, Sigmund-Freud-Straße 25, 53127 Bonn, Germany; 18 Department of Biomedical Magnetic Resonance, Otto-von-Guericke University Magdeburg, Leipziger Straße 44, 39120 Magdeburg, Germany; 19 Department of Psychiatry and Psychotherapy, University Medical Center Goettingen, University of Goettingen, Von-Siebold-Straße 5, 37075 Goettingen, Germany; 20 German Center for Neurodegenerative Diseases (DZNE), 37075 Goettingen, Von-Siebold-Str. 3a Germany; 21 iBiMED, Medical Sciences Department, University of Aveiro, Aveiro, Portugal

**Keywords:** Alzheimer’s disease, Subjective cognitive decline, Mild cognitive impairment, Longitudinal, Cerebrospinal fluid, Beta-amyloid 42, Tau, Apolipoprotein E, Magnetic resonance imaging, Positron emission tomography

## Abstract

**Background:**

Deep phenotyping and longitudinal assessment of predementia at-risk states of Alzheimer’s disease (AD) are required to define populations and outcomes for dementia prevention trials. Subjective cognitive decline (SCD) is a pre-mild cognitive impairment (pre-MCI) at-risk state of dementia, which emerges as a highly promising target for AD prevention.

**Methods:**

The German Center for Neurodegenerative Diseases (DZNE) is conducting the multicenter DZNE-Longitudinal Cognitive Impairment and Dementia Study (DELCODE), which focuses on the characterization of SCD in patients recruited from memory clinics. In addition, individuals with amnestic MCI, mild Alzheimer’s dementia patients, first-degree relatives of patients with Alzheimer’s dementia, and cognitively unimpaired control subjects are studied. The total number of subjects to be enrolled is 1000. Participants receive extensive clinical and neuropsychological assessments, magnetic resonance imaging, positron emission tomography, and biomaterial collection is perfomed. In this publication, we report cognitive and clinical data as well as apolipoprotein E (APOE) genotype and cerebrospinal fluid (CSF) biomarker results of the first 394 baseline data sets.

**Results:**

In comparison with the control group, patients with SCD showed slightly poorer performance on cognitive and functional measures (Alzheimer’s Disease Assessment Scale—cognitive part, Clinical Dementia Rating, Functional Activities Questionnaire), with all mean scores in a range which would be considered unimpaired. APOE4 genotype was enriched in the SCD group in comparison to what would be expected in the population and the frequency was significantly higher in comparison to the control group. CSF Aβ42 was lower in the SCD group in comparison to the control group at a statistical trend with age as a covariate. There were no group differences in Tau or pTau concentrations between the SCD and the control groups. The differences in all measures between the MCI group and the AD group were as expected.

**Conclusions:**

The initial baseline data for DELCODE support the approach of using SCD in patients recruited through memory clinics as an enrichment strategy for late-stage preclinical AD. This is indicated by slightly lower performance in a range of measures in SCD in comparison to the control subjects as well as by enriched APOE4 frequency and lower CSF Aβ42 concentration.

**Trial registration:**

German Clinical Trials Register DRKS00007966. Registered 4 May 2015.

## Background

Early detection of Alzheimer’s disease (AD) is crucial for the successful use of future disease-modifying therapies and for nonpharmacological interventions of secondary prevention [1]. Prodromal AD and mild cognitive impairment (MCI) due to AD have been established as predementia AD stages and are currently widely used as inclusion conditions in clinical trials [2]. However, if preservation of unimpaired cognitive function is the goal, interventions need to start at even earlier stages of AD [3]. Thus, there is a need to develop concepts for identification of individuals with AD before the clinical stages of MCI or prodromal AD are reached.

Memory services are often approached by individuals who subjectively experience decline in cognitive functioning but perform within the age, sex, and education-adjusted normal limits on standard cognitive tests. Increasing data suggest that, as a group, these individuals show slightly lower performance on challenging cognitive tasks than individuals without cognitive complaints [4, 5] and are at increased risk of cognitive decline and dementia [6]. This accounts particularly for those who have biomarker evidence for AD [7–11]. In recent consensus publications, this condition has been termed subjective cognitive decline (SCD), research criteria have been proposed, and recommendations for research studies on SCD have been provided [12, 13]. Conceptually, SCD in the presence of AD pathology may indicate the stage of first subtle decline in cognitive brain function and correspond to stage 3 of preclinical AD [14]. At this stage, SCD reflects the individual’s experience of this subtle cognitive dysfunction, which is still largely compensated [15].

SCD is highly attractive for future early interventions, because in these subjects brain function is largely preserved with intact compensatory processes, while at the same time individuals with SCD have complaints and seek medical help. In order to employ SCD in trials and prospectively in AD prevention, it is necessary to enhance knowledge about this condition with regard to cross-sectional features and longitudinal outcomes [13, 16].

This is the primary aim of the multicenter DZNE-Longitudinal Cognitive Impairment and Dementia Study (DELCODE), whose design and first baseline data are reported in this paper. The DZNE (Deutsches Zentrum für neurodegenerative Erkrankungen, German Center for Neurodegenerative Diseases) is a national research institution dedicated to molecular, clinical, epidemiological, healthcare, and nursing research on neurodegenerative diseases. It has nine operational sites in Germany, of which seven collaborate with respective local university memory centers. In total, this provides a network of 10 memory clinics. The clinical research branch of the DZNE created methodological cores for clinical assessment/neuropsychology, magnetic resonance imaging (MRI), positron emission tomography (PET), and biomaterial. These cores develop and provide standard operation procedures (SOPs) and quality control for harmonized data and material acquisition and storage across all sites.

DELCODE is a longitudinal observational study, focusing on SCD in the context of AD. The study also includes individuals with MCI and mild AD as well as control subjects without subjective or objective cognitive impairment. In addition, first-degree relatives of patients with AD dementia are enrolled as an exploratory at-risk group.

The main aims of DELCODE are: the development of a refined understanding of SCD in the context of AD; establishment of prediction models and estimates of cognitive decline in SCD; investigation of the effects of risk and protective factors on cognitive decline; and development of new disease markers. Here, we present the protocol of DELCODE and the baseline characteristics of the first 400 individuals enrolled.

## Methods

### Overall study design

DELCODE is an observational longitudinal memory clinic-based multicenter study in Germany. The participants to be enrolled are 400 subjects with SCD, 200 MCI patients, 100 AD dementia patients, 200 control subjects without subjective or objective cognitive decline, and 100 first-degree relatives of patients with a documented diagnosis of AD dementia. All patient groups (SCD, MCI, AD) are referrals, including self-referrals, to the participating memory centers. The control group and the relatives of AD dementia patients are recruited by standardized public advertisement. Ten university-based memory centers are participating, all being collaborators of local DZNE sites.

**Fig. 1:**
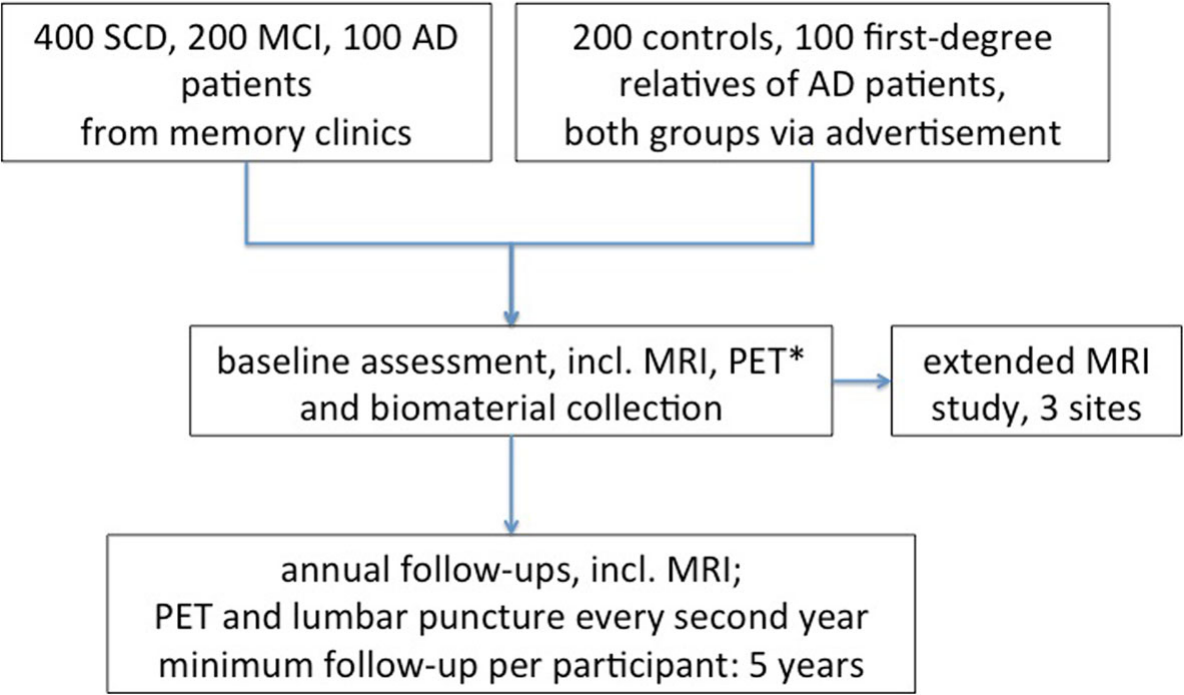
Flow chart of DELCODE. AD Alzheimer’s disease, MCI mild cognitive impairment, MRI magnetic resonance imaging, PET positron emission tomography, SCD subjective cognitive decline

The assessments within DELCODE include extended clinical and neuropsychological testing, MRI, and sampling of blood, urine, and cerebrospinal fluid (CSF). Three sites perform additional MRI on a second day. A longitudinal amyloid-PET and FDG-PET has been added with a delayed onset, for administrative reasons. Figure 1 provides an overview of the study design.

The sequence of clinical and neuropsychological examinations is harmonized across all sites. MRI and PET are scheduled according to the local conditions. The follow-up scheme of DELCODE is annual, with an individual follow-up time of 5 years and potential extension beyond.

All data and all biomaterial are stored centrally. All staff members at all sites underwent training in the respective methods to achieve high-quality data and material acquisition according to the SOP. The database and the sites are monitored centrally, including a query process. All local institutional review boards (IRB) and ethical committees approved the study protocol. In addition, the national radiation authority (Bundesamt für Strahlenschutz (BfS)) approved the PET study.

### Definition of patient groups

All patient groups (SCD, MCI, AD) were assessed clinically at the respective memory centers before entering DELCODE. The assessments include medical history, psychiatric and neurological examination, neuropsychological testing, blood laboratory work-up, and routine MRI, all according to the local standards. The Consortium to Establish a Registry for Alzheimer’s Disease (CERAD) neuropsychological test battery was applied at all memory centers to measure cognitive function. German age, sex, and education-adjusted norms of the CERAD neuropsychological battery are available online (www.memoryclinic.ch). SCD was defined by the presence of subjectively reported decline in cognitive functioning with concerns as expressed to the physician of the memory center and a test performance of better than –1.5 standard deviations (SD) below the age, sex, and education-adjusted normal performance on all subtests of the CERAD neuropsychological battery. Regarding the MCI group, only individuals with amnestic MCI were included, defined by an age, sex, and education-adjusted performance below –1.5 SD on the delayed recall trial of the CERAD word-list episodic memory tests. Both patient groups (SCD, amnestic MCI) fulfill the current research criteria for SCD [12, 13] or MCI [17], respectively. In addition, patients with mild Alzheimer’s dementia [18] and ≥ 18 points on the Mini-Mental -State Examination (MMSE) qualified for DELCODE. Note that the described procedures were all part of the clinical routine at each site and not part of DELCODE itself, but they provided the entry diagnosis of the patients for DELCODE.

The control group and the group of first-degree relatives of AD patients were recruited by identical local newspaper advertisements. In the advertisement text, individuals were explicitly sought who felt healthy and without relevant cognitive problems. All individuals who responded to the advertisement were screened by telephone with regard to SCD. The report of very subtle cognitive decline, which did not cause any concerns and was considered normal for age by the individual, was not an exclusion criterion for the control group. Further screening questions addressed other inclusion criteria and exclusion criteria (see later).

For the first-degree relatives of AD, the advertisement did not exclude those with concerns of cognitive decline. AD in the relative (parent or sibling) had to be documented by medical records.

Both the control group and the group of first-degree relatives had to achieve unimpaired cognitive performance according to the same definition as the SCD group.

All participants entered DELCODE based on either their clinical diagnosis (SCD, MCI, AD) derived from the clinical work-up or their identification as a control subject or a first-degree relative according to the procedures outlined.

Additional inclusion criteria for all groups were age ≥ 60 years, fluent German language skills, capacity to provide informed consent, and presence of a study partner. The main exclusion criteria for all groups were conditions clearly interfering with participation in the study or the study procedures, including significant sensory impairment. The following medical conditions were considered exclusion criteria: current major depressive episode, major psychiatric disorders either at baseline or in the past (e.g., psychotic disorder, bipolar disorder, substance abuse), neurodegenerative disorder other than AD, vascular dementia, history of stroke with residual clinical symptoms, history of malignant disease, severe or unstable medical condition, and clinically significant abnormalities in vitamin B12. Prohibited drugs included chronic use of psychoactive compounds with sedative or anticholinergic effects, use of anti-dementia agents in SCD, amnestic MCI, and control subjects and in healthy siblings, and investigational drugs for treatment of dementia or cognitive impairment 1 month prior to entry and for the duration of the study.

All participants gave written informed consent before inclusion in the study.

### Clinical and risk factor assessments

Within DELCODE, the clinical assessments at baseline were performed by trained study physicians. The order of examinations was fixed. All examinations were performed within 1 day and are repeated in an identical manner at the annual follow-ups.

The clinical assessments included a structured medical history, current medication, structured family history, standardized physical examination, including sensory testing, the MMSE, Clinical Dementia Rating (CDR), the 15-item short form of the Geriatric Depression Scale (GDS), the short form of the Geriatric Anxiety Inventory (GAI-SF), the Neuropsychiatric Inventory (NPI-Q), and the Functional Activities Questionnaire (FAQ). Depression and substance use were assessed in a standardized fashion according to ICD-10.

The following instruments were used for risk factor assessment: the 10-item short form of the Big Five Inventory (BFI-10), the Lifetime of Experiences Questionnaire (LEQ), the Physical Activity Scale for the Elderly (PASE), the Lubben Social Network Scale (LSNS-6), the Pittsburgh sleep quality index (PSQI), the Epworth Sleepiness Scale (ESS), and a questionnaire on REM sleep behavior disorder (RBD-Q). All participants completed the Semi-quantitative Food Frequency Questionnaire (SFFQ,) and all nondemented participants were invited to send back a more extensive nutritional questionnaire (EPIC-FFQ).

### Assessment of subjective cognitive functioning

A semi-structured interview regarding the details of SCD was administered by the study physician, who asked a series of questions regarding the presence, onset, course, and appraisal of problems with memory and other cognitive domains. This SCD interview was designed to capture the SCD-plus criteria [12], which are features of SCD that in the current state of knowledge are associated with increased likelihood of underlying AD pathology. In a separately conducted interview, study partners answered a similar set of questions, and also reported on observed cognitive changes.

In addition, the Everyday Cognition questionnaire (ECog) was applied to the participants and the study partners.

### Neuropsychological testing

Tests for the neuropsychological battery were selected in order to serve the aims of: comparability with similar ongoing studies addressing prodromal and preclinical AD (e.g., Alzheimer’s Disease Neuroimaging Initiative (ADNI)); measuring different cognitive domains (learning and memory, executive functions and processing speed, language, working memory, visuospatial functions); and including tests used in cognitive composite scores (e.g., Preclinical Alzheimer Cognitive Composite (PACC)) for tracking decline.

The test battery included the ADAScog 13, the FCSRT-IR, WMS-R Logical Memory Story A, WMS-R Digit Span, semantic fluency (animals), the oral form of the Symbol–Digit–Modalities Test (including subsequent free recall of symbols and symbol–digit pairings), Trail Making Test A and B, Clock Drawing, and Clock Copying. In addition to these established tests, two newly developed computerized tests were implemented: the Face Name Associative Recognition Test [19], and a Flanker task to assess executive control of attention [20]. The cognitive testing was performed by a trained neuropsychologist at all sites.

### Data handling and quality control

The data were captured with a web-based eCRF (webspirit, 2mt software). Edit checks, automatically ongoing during data entry, ensured completeness and plausibility of the data. The central data management coded the concomitant medication. Source data verification was performed during onsite monitoring visits. In addition, offsite monitoring was provided by the central data management unit. Inconsistencies detected during onsite, offsite, and/or medical review were queried for clarification and correction.

### Biomaterial sampling

Biomaterial sampling included blood and urine annually, and CSF in those participants who consented. CSF sampling will be offered every second year. Trained study assistants performed the collection, processing, and storage of the samples as well as shipment to the central biorepository of the DZNE according to the SOP.

Blood samples include serum samples with clotting activator (clot) and without clot, EDTA plasma and citrate plasma as well as PAXgene RNA and PAXgene DNA, EDTA whole blood, and leucocytes (during the follow-up-visits). After processing and aliquoting, all material was stored at –80 °C. Leucocytes are stored at –150 °C. Genomic DNA was isolated from EDTA whole blood samples of each participant according to the chemagic Magnetic Separation® protocol of PerkinElmer. After centrifugation, the CSF was aliquoted and stored at –80 °C.

### CSF AD biomarker assessment

AD biomarkers were determined using commercially available kits according to vendor specifications: V-PLEX Aβ Peptide Panel 1 (6E10) Kit (K15200E) and V-PLEX Human Total Tau Kit (K151LAE) (Mesoscale Diagnostics LLC, Rockville, USA), and Innotest Phospho-Tau(181P) (81581; Fujirebio Germany GmbH, Hannover, Germany). Cutoff values for normal and abnormal concentrations of Aβ42 (<496 pg/ml) and for the ratio Aβ42/Aβ40 (< 0.09) were derived from the literature, which applied the respective assays [21]. We used cutoff values established locally (Bonn) based on clinical nonimpaired control samples for Tau (> 470 pg/ml) and pTau (> 57 pg/ml).

In addition, we calculated the Hulstaert formula to define an abnormal Aβ42/Tau ratio [22]:

$$ \mathrm{A}\upbeta 42/\left[240+1.18\times \mathrm{Tau}\right]<1. $$

This formula has been shown to be a very robust indicator of AD pathology in several independent studies (e.g., [23]). The overall CSF sampling rate in DELCODE is around 50%. Here, we report data on 144 participants. The subsample of participants with CSF differed neither in demographic variables (sex, age, years of education) nor the MMSE from the subsample without CSF.

### APOE genotyping

Genotypes for rs7412 and rs429358, the single nucleotide polymorphisms (SNPs) defining the ε-2, ε-3, and ε-4 alleles of APOE, were genotyped using commercially available TaqMan® SNP Genotyping Assay (ThermoFisher Scientific). Both SNP assays were amplified on genomic DNA using a StepOnePlus Real-Time PCR System (ThermoFisher Scientific). Visual inspection of cluster formation was performed for each SNP before genotype data were further used to define ε-2, ε-3, and ε-4 alleles in each sample.

### Magnetic resonance imaging

MRI data were acquired at nine scanning sites. All sites operate Siemens scanners, including three TIM Trio systems, four Verio systems, one Skyra system, and one Prisma system.

The standard DELCODE MR protocol included a structural T1-weighted image, a resting state fMRI (including IR-EPI and a field map), a T2-weighted structural scan optimized for volumetric assessment of the medial temporal lobe acquired in oblique coronal orientation perpendicular to the longitudinal axis of the hippocampus, a task fMRI (scene novelty and encoding task), and a quantitative susceptibility weighted image. This protocol was used in eight out of the nine scanning sites. One of the sites did not have the provision to conduct the task fMRI and instead conducted a diffusion tensor imaging (DTI) protocol. At three sites participants also underwent an optional second day of scanning with DTI, a task fMRI to assess object and scene processing and mnemonic discrimination, and a T1-weighted FLASH sequence optimized to image the locus coeruleus.

For task fMRI, all sites were equipped with a high-resolution (1280 Px × 800 Px) 30-inch MR-compatible LCD screen (“Medres Optostim”). All monitors were calibrated and configured to maintain the distance, luminance, color, and contrast constant across sites. Responses during task fMRI were recorded at all sites with MR-compatible response buttons (CurrentDesign). All participants underwent vision correction with MR-compatible goggles (Medigoogle; Cambridge Research Systems) according to the same SOP for all MRI sites. Task fMRI scenario files were controlled with Presentation (Neurobehavioral Systems).

For quality assurance (QA) and assessment, the following steps were taken. The DZNE imaging network, headed by the Magdeburg DZNE site (iNET), qualified each MRI site with a traveling head measurement prior to the start of the study. DZNE iNET then provided every site with detailed SOPs for the implementation of each protocol. All radiographers who operate MRIs in the study underwent centralized training to implement the SOPs (i.e., subjects’ positioning in the MRI scanner, sequence preparation steps, image angulation, task-fMRI visual acuity checks and correction, participant instruction, and testing).

A small MRI-phantom built and designed by the American College of Radiology (ACR) is used to monitor the performance of the MR systems on a weekly basis. The phantom images are analyzed according to a published protocol [24]. A custom-built holder was designed to maximize reproducibility in phantom positioning across all nine MRI sites.

For QA, every scan underwent a quality check for SOP conformity and scan quality by the DZNE iNET team (Magdeburg). To establish inclusion/exclusion criteria based on data-driven quantitative metrics, a Bayesian-based strategy is being developed which will use the current manual/semi-automatic QA information for training after processing the images using the pcp-qa package (www.preprocessed-connectomes-project.org/quality-assessment-protocol/)). Details on individual MR sequences and the fMRI experiments will be reported together with results in subsequent publications.

Baseline MRI scans of 367 individuals were obtained from the initial 400 participants reported here. In addition, 117 datasets of the extended protocol were acquired.

### Positron emission tomography

The PET protocol is the multicenter substudy of DELCODE that focuses on SCD subjects only. The participants will be scanned twice within 2 years of follow-up with the PET tracers ^18^F-florbetaben (FBB; Neuraceq™; Piramal Imaging) and ^18^F-fluordesoxyglucose (FDG). Approval for DELCODE PET by the Federal Radiation Protection Authority (Bundesamt für Strahlenschutz (BfS)) and ethical approval at all participating sites were obtained. SOPs were generated to harmonize data acquisition across sites. These include tight time frames between clinical/neuropsychological assessments and FDG-PET, standardized dosing regimens, and scan positioning, among other aspects. PET-CT is currently used in seven cooperating sites; one site currently uses PET-MR. Regular quality assessments were implemented, including yearly Hoffmann 3D brain phantom measurements at all participating sites. Phantom data will also be used to harmonize image resolutions across scanners/sites [25]. Basic FBB data analysis will include region of interest (ROI)-based analysis using definitions of standard value uptake ratios in PMOD (PMOD TECHNOLOGIES LLC, Zürich, Switzerland), with the cerebellar cortex as the reference region [26]. Assessments of amyloid deposition are computed for regional and composite ROIs. FDG data will be analyzed with both voxel-based and ROI-based methods. PET data will be reported in subsequent publications. Since the protocol has only been started with a substantial delay, 25 amyloid-PET scans and 16 FDG-PET scans were obtained in association with the 400 baseline datasets of this report.

### Statistical analysis

The main dependent variables of DELCODE are measures of cognition and function. Future analyses will employ several statistical approaches, including but not limited to regression models for the identification of predictors of decline.

All statistical analyses for the present report were performed with SPSS-23 for Windows. Here, we focus on descriptive statistics and differences between the groups on key demographic, clinical, and neuropsychological variables as well as APOE genotype and CSF-biomarker data. Regarding the latter, we report both continuous and dichotomized values (i.e., “biomarker positive vs negative” subjects, classified according to the cutoff values reported earlier). Differences between groups were tested with a series of ANOVAs for continuous variables and chi-square tests for categorical variables, respectively. We report the statistics for the overall group effect (*F*-statistic/omnibus chi-square test with *p* value) and indicate significant single contrasts of each group compared to the control group, respectively (based on post-hoc *t* tests or chi-square tests). For those CSF parameters with group differences particularly between SCD patients and controls, we calculated the effect size and also report age-adjusted results based on ANCOVA or logistic regression statistics, respectively. Because this report is descriptive rather than hypothesis driven and is based on only a subsample of the prospected baseline sample of DELCODE, we report all *p* values in an exploratory way, unadjusted for multiple testing.

## Results

For this publication, the data of the first 400 enrolled participants were cleaned and exported from the database. Six datasets were excluded from the analysis due to implausible scores on key variables with regard to the diagnostic group, which could not be resolved by the respective sites. Thus, 394 individuals are included in the present report. Basic demographic and clinical characteristics, cognitive performance in main tests, CSF biomarkers, and APOE genotype are presented in Table 1.

**Table 1:** Description of the sample

	CO (*n* = 141)	SCD (*n* = 126)	MCI (*n* = 65)	AD (*n* = 40)	Relatives of AD patients (*n* = 22)	*F* value/χ^2^ value
Age (years), mean (SD)	68.6 (5.1)	71.4 (5.7)***	72.8 (5.2)***	72.8 (7.1)**	64.8 (4.5)**	15.4, *p* > 0.001
Sex (female), *n* (%)	83 (58.9)	65 (51.6)	25 (38.5)**	23 (57.5)	12 (54.5)	7.9, n.s.
Education (years), mean (SD)	15.0 (2,7)	14.7 (3.1)	14.1 (3.1)	13.2 (3.2)**	13.7 (2.6)*	3.78, *p* = 0.005
MMSE, mean (SD)	29.4 (0.9)	29.1 (1.0)	28.0 (1.6)***	23.6 (3.3)***	29.0 (1.2)	135, *p* < 0.001
CDR, mean (SD)	0.0 (0.0)	0.2 (0.2)***	0.5 (0.1)***	0.8 (0.2)***	0.1 (0.2)	216, *p* < 0.001
CDR-SOB, mean (SD)	0.0 (0.1)	0.4 (0.4)***	1.6 (1.2)***	4.7 (1.3)***	0.4 (1.0)	343, *p* < 0.001
ADAScog 13, mean (SD)	5.6 (3.3)	7.0 (3.9)**	15.3 (6.3)***	29.1 (8.1)***	8.0 (5.2)	220, *p* < 0.001
FAQ, mean (SD)	0.1 (0.4)	0.8 (1.2)***	3.5 (4.4)***	11.8 (6.2)***	0.4 (1.0)	154, *p* < 0.001
GDS, mean (SD)	0.7 (1.4)	1.9 (1.7)***	1.8 (1.6)***	2.1 (1.5)***	1.1 (1.3)	12.5, *p* < 0.001
APOE genotype	(*n* = 131)	(*n* = 114)	(*n* = 60)	(*n* = 36)	(*n* = 19)	
APOE4 genotype of all APOE, *n* (%)	23 (17.6)	37 (32.5)**	22 (36.7)**	25 (69.4)***	8 (42.1)*	37.3, *p* < 0.001
CSF biomarkers	(*n* = 50)	(*n* = 45)	(*n* = 35)	(*n* = 18)	(*n* = 6)	
Aβ42 (pg/ml), mean (SD)	890 (323)	748 (329)*	630*** (303)	353 (100)***	684 (415)	11.1, *p* < 0.001)
Aβ42 < 496 pg/ml, *n* (%)	5 (10.0)	9 (20.0)	16 (45.7)***	18 (100)***	2 (33.3)	54.9, *p* < 0.001
Aβ42/Aβ40, mean (SD)	0.099 (0.022)	0.093 (0.026)	0.077 (0.031)**	0.046 (0.015)***	0.077 (0.036)	16.0, *p* < 0.001
Aβ42/Aβ40 < 0.09, *n* (%)	14 (28.0)	17 (37.8)	21 (60.0)**	17 (94.4)***	4 (66.7)	28.3, *p* < 0.001
tTau (pg/ml), mean (SD)	359 (159)	366 (157)	502** (224)	742 (309)***	370 (108)	15.6, *p* < 0.001
tTau > 470 pg/ml, *n* (%)	8 (16.0)	11 (24.4)	16 (45.7)**	15 (83.3)***	1 (16.7)	31.9, *p* < 0.001
pTau 181 (pg/ml), mean (SD)	51 (20)	51 (24)	67 (33)*	91 (43)***	53 (16)	9.3, *p* < 0.001
pTau 181 > 57 pg/ml, *n* (%)	15 (30.0)	14 (31.1)	20 (57.1)*	15 (83.3)***	3 (50.0)	20.9, *p* < 0.001
Aβ42/Tau ratio, Hulstaert formula, mean (SD)	1.36 (0.38)	1.15 (0.47)*	0.84 (0.49)***	0.34 (0.14)***	1.03 (0.63)	22.0, *p* < 0.001
Abnormal Aβ42/Tau ratio, Hulstaert formula, *n* (%)	7 (14.0)	16 (35.6)**	23 (65.7)***	18 (100)***	2 (33.3)	46.7, *p* < 0.001

Post-hoc unadjusted *p* value in comparison to the control group: *p < 0.05, **p < 0.01, ***p < 0.001

*Aβ42* beta-amyloid 42, *AD* Alzheimer’s disease, *ADAScog 13* Alzheimer’s Disease Assessment Scale—cognitive part, *APOE* apolipoprotein E, *CDR* Clinical Dementia Rating, *CDR-SOB* Clinical Dementia Rating—sum of boxes, *CO* control group, *CSF* cerebrospinal fluid, *FAQ* Functional Activities Questionnaire, *GDS* Geriatric Depression Scale, *MCI* mild cognitive impairment, *MMSE* Mini-Mental -State Examination, *n.s.* not significant, *SCD* subjective cognitive decline, *SD* standard deviation

### Demographic, cognitive, and clinical data

The control group was slightly younger than the patient group and the group of relatives. The amnestic MCI group included a lower number of female patients. The AD group and the group of first-degree relatives had a slightly lower number of years of education in comparison to the other groups.

The MMSE did not differ between the SCD group and the control group and it did not differ between the first degree-relatives and the control group, while the MCI and AD groups showed lower scores than the controls. Interestingly, ADAScog, CDR total score, CDR sum of boxes (CDR-SOB), and the FAQ score were significantly higher in the SCD group in comparison with the control group, indicating subtle worse performance. The scores in the SCD group were similar to those of the first-degree relative group, which were, however, not significantly different from control group due to the limited sample size. The GDS scores were also higher in all patient groups in comparison with the control group.

### Biological markers

The frequency of the APOE4 genotype was significantly higher in the amnestic MCI, AD, and first-degree relative groups in comparison to the control group. It was also significantly higher in the SCD group in comparison with the control group (32.5% vs 17.6%). The amnestic MCI and AD groups showed the typical AD pathology-type CSF profile with reduced Aβ42 and increased Tau and pTau concentrations as well as lower Aβ42/Tau ratio scores (Hulstaert formula). The SCD group showed lower Aβ42 concentration than the control group with an effect size of Cohen’s *d* = 0.44. Including age as a covariate revealed a difference at a statistical trend level of *p* = 0.084, while age did not have a significant effect (*p* = 0.51). The number of SCD patients with an Aβ42 level below the predefined cutoff value was numerically higher than in the control group (20% vs 10%). Similarly, the number of SCD patients with an Aβ42/Aβ40 ratio below the prespecified cutoff value was numerically higher in the SCD group than in the control group (37.8% vs 28%). There were no meaningful differences in Tau and pTau concentration between the SCD and the control groups. Analysis of the Aβ42/Tau ratio (Hulstaert formula) revealed a significant difference between the SCD and the control groups with lower scores in the SCD group, indicative of AD pathology (Cohen’s *d* = 0.5). This effect was not significant after controlling for age (*p* = 0.11), while age had a significant effect (*p* = 0.037). The number of subjects in the SCD group who had a Hulstaert score below the AD-type cutoff value was significantly higher than in the control group (35.6% vs 14%). A logistic regression analysis with age as an additional predictor revealed an odds ratio (OR) of 2.62 (CI 0.917–7.487, *p* = 0.072) for the diagnostic group (SCD vs CO) and of 1.103 (CI 0.995–1.223, *p* = 0.061) for age per year.

## Discussion

In this report, we describe the DELCODE protocol and basic results of the first 394 baseline datasets. The main focus of the study is on SCD as a pre-MCI at-risk state of AD dementia.

In comparison to the control group, the SCD group showed slightly worse performance on the ADAScog. However, the mean score of 7.0 points on the ADAScog in the SCD group still reflects performance in the unimpaired range. The mean ADAScog score of the MCI group was 15.3. The global CDR score and the CDR-SOB were also slightly higher in the SCD group (mean 0.2 and 0.4, respectively). Finally, the FAQ, which indicates the performance on activities of daily living, was slightly higher in the SCD group in comparison to the controls. The mean score of 0.8 in the SCD group, however, is clearly below a score of 6 points indicating IADL impairment at the level of dementia [27]. Overall these data indicate subtle lower cognitive and functional performance in the SCD group in comparison to the control group. Importantly, neither the cognitive nor the functional scores would be considered outside the normal range in the SCD group and are clearly distinct from the performance of the amnestic MCI group. These findings support the concept of SCD (at least in memory clinics) corresponding to the subtle decline in performance, which may correspond to the late stage of preclinical AD [12, 14].

We observed a frequency of APOE4 allele carriers of 32.5% in the SCD group in comparison to a significantly lower proportion of E4 carriers in the control group (17.6%), indicating APOE4 enrichment. The frequency of the APOE4 allele in the general population in Germany has been reported to be 14.5% [28].

The CSF biomarkers indicated a lower Aβ42 concentration in the SCD group in comparison with the control group. The effect was of medium magnitude (*d* = 0.44) and reached a statistical trend level toward significance after controlling for age. We did not observe increased Tau or pTau in the SCD group in comparison to the control group. This is an intriguing finding, suggesting that first subtle symptoms may already occur at the stage of Aβ42 deposition only, without significant neurodegeneration. This sequence has recently been proposed in a conceptual model of SCD in the context of AD [16].

However, there was also a higher rate of SCD individuals with suggestive AD pathology according to the Hulstaert score, which integrates Aβ42 and Tau into a ratio and has been reported as a powerful predictor for conversion from MCI [e.g., 23] and also SCD [11] to AD dementia. The OR of SCD with regard to evidence of AD pathology according to this score was 2.62 with adjustment for age, which also showed a trend toward significance. In addition, the number of SCD patients with abnormal Hulstaert scores in the present sample (35.6%) is remarkably similar to the rate of 34.1% observed in a fully independent sample of memory clinic patients with SCD from the German Dementia Competence Network [11].

The present report has limitations. It comprises only the first 394 baseline datasets of DELCODE, while the project aims at including 1000 individuals. This limits statistical power, particularly in the biomarker subgroups. At the present state, data are only exploratory and not generalizable. PET data are not yet available at a sufficient number for statistical analyses. Longitudinal data are also not available for the present analysis. Analyses of all assessments, brain imaging, and further biomaterial studies will be reported in further publications.

## Conclusions

We found support for the model that SCD is associated with very mildly reduced cognitive and functional performance in comparison with individuals without SCD in our multicenter memory clinic study. There was also evidence for an enrichment of APOE4 genotype and for Aβ-positive individuals in the SCD group, while there was no evidence for increased Tau pathology. These preliminary results support the concept that SCD may serve as an enrichment strategy for AD and may correspond to late-stage preclinical AD, indicating its first symptomatic manifestation.

## Abbreviations

3D: Three dimensional
ACR: American College of Radiology
AD: Alzheimer’s disease
ADAScog 13: Alzheimer’s Disease Assessment Scale—cognitive part
ADNI: Alzheimer’s Disease Neuroimaging Initiative
ANOVA: Analysis of variance
APOE: Apolipoprotein E
BFI-10: Big Five Inventory
BfS: Bundesamt für Strahlenschutz
CDR: Clinical Dementia Rating
CDR-SOB: Clinical Dementia Rating—sum of boxes
CERAD: Consortium to Establish a Registry for Alzheimer’s Disease
CSF: Cerebrospinal fluid
DELCODE: DZNE-Longitudinal Cognitive Impairment and Dementia Study
DNA: Deoxyribonucleic acid
DTI: Diffusion tensor imaging
DZNE: German Centre for Neurodegenerative Diseases (Deutsches Zentrum für Neurodegenerative Erkrankungen)
ECog: Everyday Cognition questionnaire
eCRF: Electronic case report form
EDTA: Ethylenediaminetetraacetic acid
EPIC-FFQ: EPIC-Food Frequency Questionnaire
ESS: Epworth Sleepiness Scale
FAQ: Functional Activities Questionnaire
FBB: ^18^F-Florbetaben
FCSRT-IR: Free and cued selective reminding test—immediate recall
FDG: ^18^F-Fluordesoxyglucose
FLASH: Fast low-angle shot
fMRI: Functional magnetic resonance imaging
GAI-SF: Geriatric Anxiety Inventory—short form
GDS: Geriatric Depression Scale
ICD-10: International Classification of Diseases
iNET: Imaging network
IRB: Institutional review board
LCD: Liquid crystal display
LEQ: Lifetime of Experiences Questionnaire
LSNS-6: Lubben Social Network Scale
MCI: Mild cognitive impairment
MMSE: Mini-Mental -State Examination
MRI: Magnetic resonance imaging
NPI-Q: Neuropsychiatric Inventory
PACC: Preclinical Alzheimer Cognitive Composite
PASE: Physical Activity Scale for the Elderly
PAXgene: PreAnalyticX™
PCR: Polymerase chain reaction
PET: Positron emission tomography
PET-CT: Positron emission tomography–computer tomography
PET-MR: positron emission tomography–magnetic resonance
PMOD: Peripheral Module
PSQI: Pittsburgh sleep quality index
QA: Quality assurance
RBD-Q: Questionnaire on REM sleep behavior disorder
RNA: Ribonucleic acid
ROI: Region of interest
SCD: Subjective cognitive decline
SD: Standard deviation
SFFQ: Semi-quantitative Food Frequency Questionnaire
SNP: Single nucleotide polymorphism
SOP: Standard operation procedure
SPSS-23: Statistical Package for the Social Sciences—23rd edition
WMS-R: Wechsler Memory scale—revised
